# Istradefylline Mitigates Age-Related Hearing Loss in C57BL/6J Mice

**DOI:** 10.3390/ijms22158000

**Published:** 2021-07-27

**Authors:** Min Shin, Madhavi Pandya, Kristan Espinosa, Ravindra Telang, Jordi Boix, Peter R. Thorne, Srdjan M. Vlajkovic

**Affiliations:** 1 Department of Physiology and The Eisdell Moore Centre, Faculty of Medical and Health Sciences, The University of Auckland, Private Bag, Auckland 1142, New Zealand; m.shin@auckland.ac.nz (M.S.); m.pandya@auckland.ac.nz (M.P.); k.espinosa@auckland.ac.nz (K.E.); r.telang@auckland.ac.nz (R.T.); pr.thorne@auckland.ac.nz (P.R.T.); 2 Centre for Brain Research, Faculty of Medical and Health Sciences, The University of Auckland, Private Bag, Auckland 1023, New Zealand; j.boix-i-coll@auckland.ac.nz

**Keywords:** age-related hearing loss, cognitive function, adenosine A_2A_ receptor, istradefylline, otoprotection, C57BL/6 mouse

## Abstract

Age-related hearing loss (ARHL) is the most common sensory disorder among older people, and yet, the treatment options are limited to medical devices such as hearing aids and cochlear implants. The high prevalence of ARHL mandates the development of treatment strategies that can prevent or rescue age-related cochlear degeneration. In this study, we investigated a novel pharmacological strategy based on inhibition of the adenosine A_2A_ receptor (A_2A_R) in middle aged C57BL/6 mice prone to early onset ARHL. C57BL/6J mice were treated with weekly istradefylline (A_2A_R antagonist; 1 mg/kg) injections from 6 to 12 months of age. Auditory function was assessed using auditory brainstem responses (ABR) to tone pips (4–32 kHz). ABR thresholds and suprathreshold responses (wave I amplitudes and latencies) were evaluated at 6, 9, and 12 months of age. Functional outcomes were correlated with quantitative histological assessments of sensory hair cells. Cognitive function was assessed using the Morris water maze and the novel object recognition test, and the zero maze test was used to assess anxiety-like behaviour. Weekly injections of istradefylline attenuated ABR threshold shifts by approximately 20 dB at mid to high frequencies (16–32 kHz) but did not improve ABR suprathreshold responses. Istradefylline treatment improved hair cell survival in a turn-dependent manner, whilst the cognitive function was unaffected by istradefylline treatment. This study presents the first evidence for the rescue potential of istradefylline in ARHL and highlights the role of A_2A_R in development of age-related cochlear degeneration.

## 1. Introduction

Age-related hearing loss (ARHL), or presbyacusis, is the most common sensory deficit affecting over 65% of adults above 60 years of age [1]. ARHL is a multifaceted pathology resulting from genetic and environmental causes. Genetic factors determine the rate and extent of cochlear degeneration, but the severity of hearing loss is also influenced by previous ear diseases, chronic illnesses, cumulative noise exposure, use of ototoxic drugs, and lifestyle [1,2]. ARHL is characterised by progressive decline in hearing sensitivity, impaired speech discrimination and sound localisation, and central auditory processing deficits [3,4]. ARHL can lead to social isolation and depression but may also contribute to more generalised cognitive impairment. Even mild levels of hearing loss increase the long-term risk of cognitive decline and dementia [4,5].

In humans, age-related cochlear pathology includes loss of sensory hair cells and primary auditory neurons and degenerative processes in the cochlear lateral wall (stria vascularis) that can result in progressive hearing impairment which begins at the highest frequencies [6]. Reduction in vascularisation in the stria vascularis, cumulative oxidative stress, low-grade inflammation, impaired mitochondrial quality control, and mitochondrial DNA damage are the pathological processes with a critical role in the development of ARHL [7,8,9,10]. Our current understanding of the mechanisms of ARHL is largely derived from animal models. The C57BL/6 mouse is the most established model of accelerated ARHL, with hearing loss commencing at about 6 months of age and becoming severe by 12 months of age [11,12,13]. These mice also show an age-related decline in cognitive function [14].

Treatment options for hearing loss in general are limited to medical devices, such as hearing aids and cochlear implants; however, these have significant limitations because the ear still remains damaged. Currently, there are no clinical therapies that can rescue the dying sensory hair cells and auditory neurons or regenerate these cells. We are, therefore, focussed on developing therapies that can prevent as well as repair injury to the inner ear structures and, thus, prevent functional deficits.

We have previously identified the adenosine A_1_ receptor (A_1_R) as one of the most promising targets for the treatment of acute noise-induced cochlear injury and other forms of sensorineural hearing loss [15,16,17,18]. Systemic or local administration of A_1_R agonists in the post-exposure period leads to the improvement of auditory thresholds, reduced expression of oxidative stress markers, and increased survival of sensory hair cells in the noise-exposed cochlea [15,16]. A_1_R agonism can also mitigate cisplatin and aminoglycoside ototoxicity [17,18]. This supports our view that A_1_R is an important regulator of cochlear survival in stress and injury [19]. However, the use of A_1_R agonists in chronic conditions such as ARHL is limited by systemic cardiovascular side effects and effect inversion after prolonged treatment [20].

The adenosine A_2A_ receptor (A_2A_R) also has an important role in neuroprotection, but as a negative regulator of brain injury; hence, the clinical focus is on A_2A_R inhibition. A_2A_R antagonists confer neuroprotection in animal models of stroke, Parkinson’s and Alzheimer’s disease, and traumatic brain injury, most likely by controlling neuroinflammation, excitotoxic neuronal damage, and synaptopathy [20,21]. Similarly, studies in mice lacking the *A_2A_R* (*adora2a*) gene demonstrated reduced neuronal injury after occlusion of the middle cerebral artery and neuroprotection in several models of neurodegenerative diseases [21,22]. Our studies on adenosine A_2A_R-deficient mice demonstrated better preservation of the sensory hair cells, afferent synapses, and primary auditory neurons in the noise-exposed cochlea relative to wildtype mice [23]. In addition, we have shown that istradefylline, an A_2A_R antagonist, can mitigate excitotoxic injury caused by administration of glutamate receptor agonists NMDA and kainic acid in organotypic tissue cultures of the neonatal rat cochlea [24]. In that study, istradefylline treatment reduced deafferentation of sensory hair cells and improved the survival of afferent synapses after excitotoxic injury [24]. This raised the possibility that A_2A_R inhibition could be used in therapeutic management of ARHL to improve the survival of sensorineural tissues in the ageing cochlea.

In the present study, we investigated the effect of the A_2A_R antagonist istradefylline on the development of age-related cochlear degeneration in a mouse model of accelerated ARHL (C57BL/6 mouse). We also investigated cognitive function in mice treated with istradefylline to exclude neurotoxic side effects of treatment. Istradefylline is a clinically and FDA-approved drug that has been used in conjunction with L-DOPA to alleviate motor symptoms of Parkinson’s disease [25,26,27]. It has high affinity and selectivity for adenosine A_2A_R and excellent safety profile in human studies [20,27]. The present study uncovers a potentially important protective role of istradefylline in the development of age-related cochlear degeneration in a mouse model of accelerated ARHL.

## 2. Results

### 2.1. Body Weights and Auditory Brainstem Responses in Mice Treated with Istradefylline 

**Figure 1:**
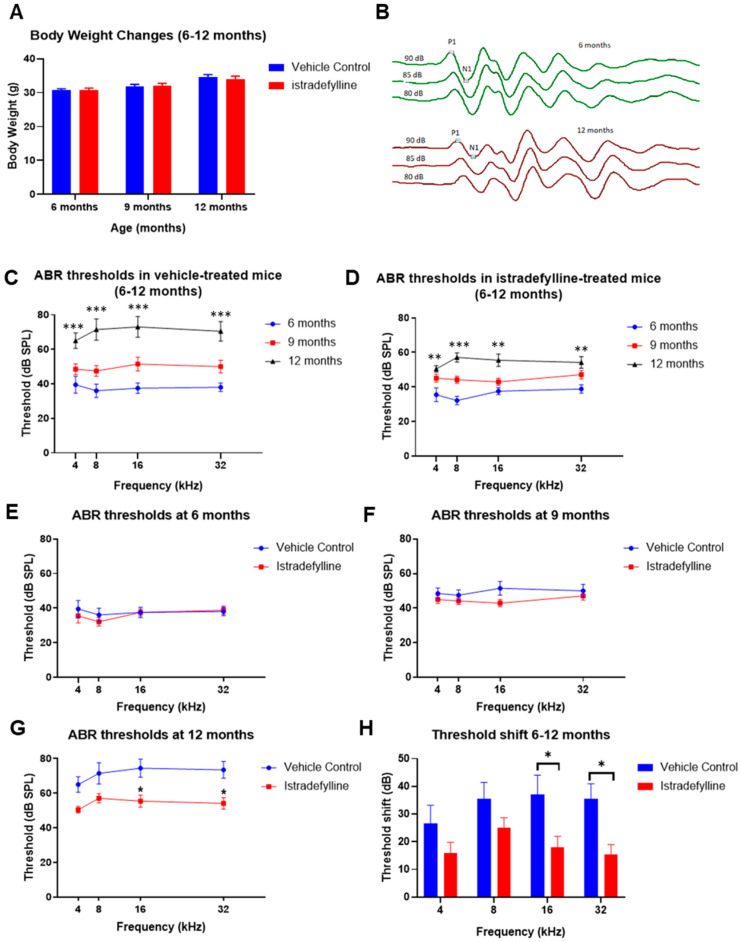
(**A**) Body weight changes in C57BL/6J mice from 6 to 12 months of age; (**B**) representative traces of ABR wave I at 6 months (green) and 12 months (red) recorded at 16 kHz at suprathreshold intensities. The peak (P1) and trough (N1) of ABR wave I are shown; (**C**) age-related increase in ABR thresholds for tone pips (4–32 kHz) in control vehicle-treated and (**D**) istradefylline-treated mice; (**E**) comparison of ABR thresholds at 6 months; (**F**) 9 months; (**G**) 12 months; (**H**) ABR threshold shifts in istradefylline- and control vehicle-treated mice calculated as the difference between ABR thresholds at 12 months and baseline ABR thresholds at 6 months. Data are expressed as means ± SEM. Vehicle control, *n* = 13; Istradefylline, *n* = 12. * *p* < 0.05; ** *p* < 0.01; *** *p* < 0.001. Two-way ANOVA followed by Holm–Sidak post hoc test.

Body weights slightly increased over time in all mice and were similar in istradefylline- and control vehicle-treated groups at 6, 9, and 12 months of age (Figure 1A). Auditory brainstem responses (ABR) were used to measure auditory thresholds in all animals and representative ABR traces (baseline and final) are shown in Figure 1B. Age progression from 6 to 12 months caused gradual elevation of ABR thresholds in both control and istradefylline-treated animals (Figure 1C,D). Whilst ABR thresholds were similar in istradefylline-treated and control animals at 6 and 9 months of age (Figure 1E,F), at 12 months, ABR thresholds were significantly (*p* < 0.05) reduced in the istradefylline group relative to controls (Figure 1G). In the vehicle-treated group, the average threshold shifts (calculated as the frequency-specific threshold differences at 12 months and 6 months of age) were between 26 and 37 dB across the test frequencies (4–32 kHz), with the greatest shift observed in the mid to high frequencies (Figure 1H). ABR threshold shifts were significantly reduced in istradefylline-treated animals at 16 kHz (*p* = 0.034) and 32 kHz (*p* = 0.023) by 19 and 20 dB, respectively. At lower frequencies, the threshold shifts were also slightly improved, but this was not statistically significant (*p* = 0.44 at 4 kHz; *p* = 0.46 at 8 kHz).

### 2.2. Input–Output Functions

To further investigate the otoprotective effects of istradefylline treatment, three test frequencies (4 kHz, 16 kHz, and 32 kHz) were selected as representative of the low, mid, and high frequency regions of the cochlea, respectively. The amplitudes and latencies of wave I were analysed at suprathreshold intensities (80, 85, and 90 dB SPL).

**Figure 2:**
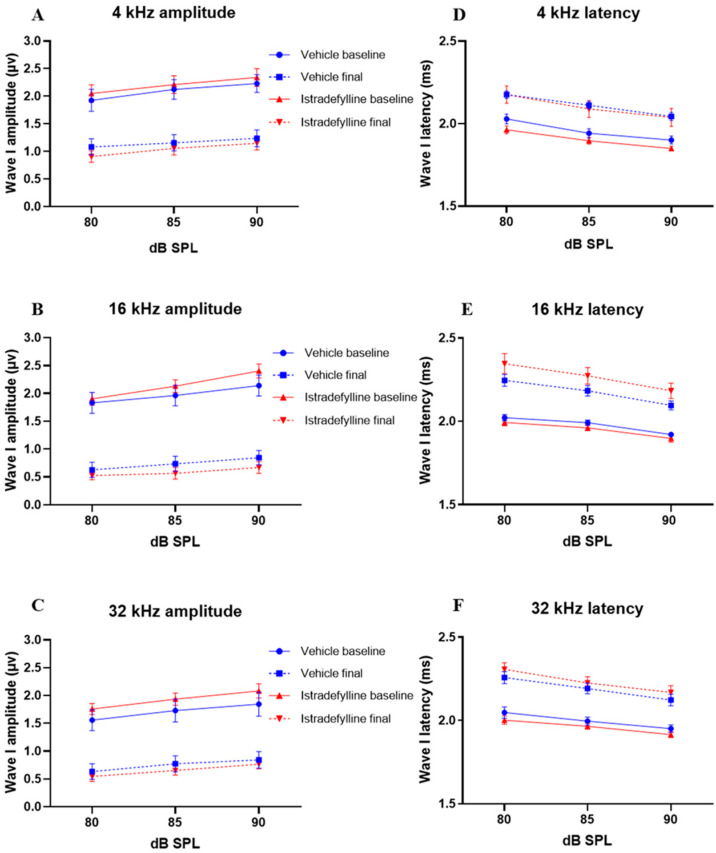
Average baseline (6 months) and final (12 months) ABR wave I amplitudes (**A**–**C**) and latencies (**D**–**F**) at 4, 16, and 32 kHz. Amplitudes and latencies were recorded at suprathreshold intensities (80–90 dB SPL) in mice treated with istradefylline or drug vehicle solution. Solid lines represent baseline amplitudes/latencies and dashed lines represent final amplitudes/latencies. Data presented as mean ± SEM. Vehicle control, *n* = 13; istradefylline, *n* = 12.

At 6 months (baseline), ABR wave I amplitudes and latencies were similar in control and istradefylline-treated animals at each test frequency. At 12 months of age, wave I amplitudes decreased, and latencies increased in all animals (Figure 2). The control group showed an average 45–61% reduction in wave I amplitudes at 90 dB SPL across all frequencies, with 16 kHz as the most affected frequency. The istradefylline-treated mice exhibited similar reduction in ABR wave I amplitudes as the control group at all test frequencies (Figure 2A–C).

Ageing also increased wave I latencies in the control group by 7.5–9%, and a similar increase in latencies was also observed in istradefylline-treated animals across all test frequencies (Figure 2D–F).

### 2.3. Hair Cell Survival

For hair cell counts, cochlear whole mounts were divided into three segments: the apical, middle, and basal (0–30%, 30–65%, and 65–100% distance from the apex, respectively). Hair cell loss was, then, compared between vehicle- and istradefylline-treated animals at 12 months of age. As expected, we observed turn-related differences in hair cell loss, with the most heavily affected region being the high frequency region of the cochlea (the basal turn).

**Figure 3:**
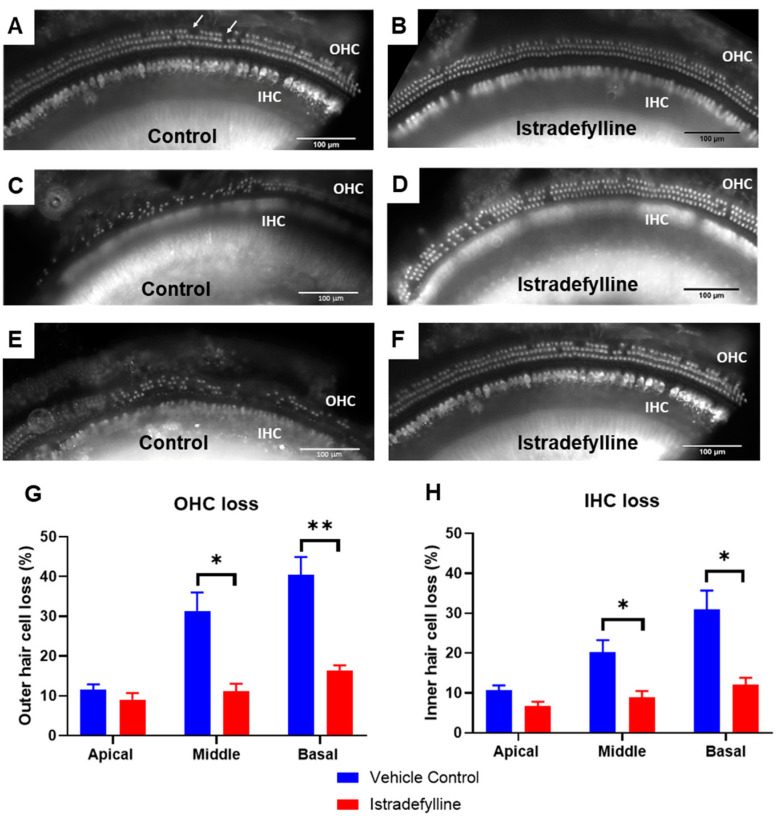
Representative images of the organ of Corti in the apical turn of the cochlea in (**A**) control vehicle-treated and (**B**) istradefylline-treated mice. Anti-Myosin VIIa antibody was used to label sensory hair cells. White arrows point at missing hair cells. Representative images of the organ of Corti are also shown in the middle (**C**,**D**) and basal (**E**,**F**) turns of the cochlea. (**G**) OHC loss (%) in the apical, middle, and basal cochlear turns in vehicle- and istradefylline-treated mice. (**H**) IHC loss (%). Data presented as mean ± SEM. Vehicle control, *n* = 13; istradefylline, *n* = 12. * *p* < 0.05, ** *p* < 0.01. Two-way ANOVA followed by Holm–Sidak post hoc test.

The middle and basal turns demonstrated a greater loss of OHCs compared to IHCs in both vehicle- and istradefylline-treated animals (see the representative images of the organ of Corti in all three cochlear turns, Figure 3A–F). The average OHC loss for the vehicle-treated animals was 31.4 ± 4.6% and 40.5 ± 4.4% for the middle and basal turns, respectively (Figure 3G). Treatment with istradefylline reduced OHC loss to an average of 11.1 ± 1.9% in the middle turn (*p* = 0.016) and 16.4 ± 1.2% in the basal turn (*p* = 0.007) (Figure 3G). Similarly, the IHC loss was significantly reduced with istradefylline treatment, from 20.3 ± 3.0% to 8.9 ± 1.6% (*p* = 0.023) in the middle turn and from 30.9 ± 4.8% to 12 ± 1.8% (*p* = 0.019) in the basal turn of the cochlea (Figure 3H). In the apical turn, the survival of sensory hair cells (IHC and OHC) was similar in vehicle- and istradefylline-treated mice (Figure 3G,H).

Overall, the basal turn was most affected in ageing mice and showed the greatest hair cell loss. Treatment with istradefylline induced significant improvement in IHC and OHC survival in both middle and basal turns of the cochlea (Figure 3).

### 2.4. Behavioural Studies

**Figure 4:**
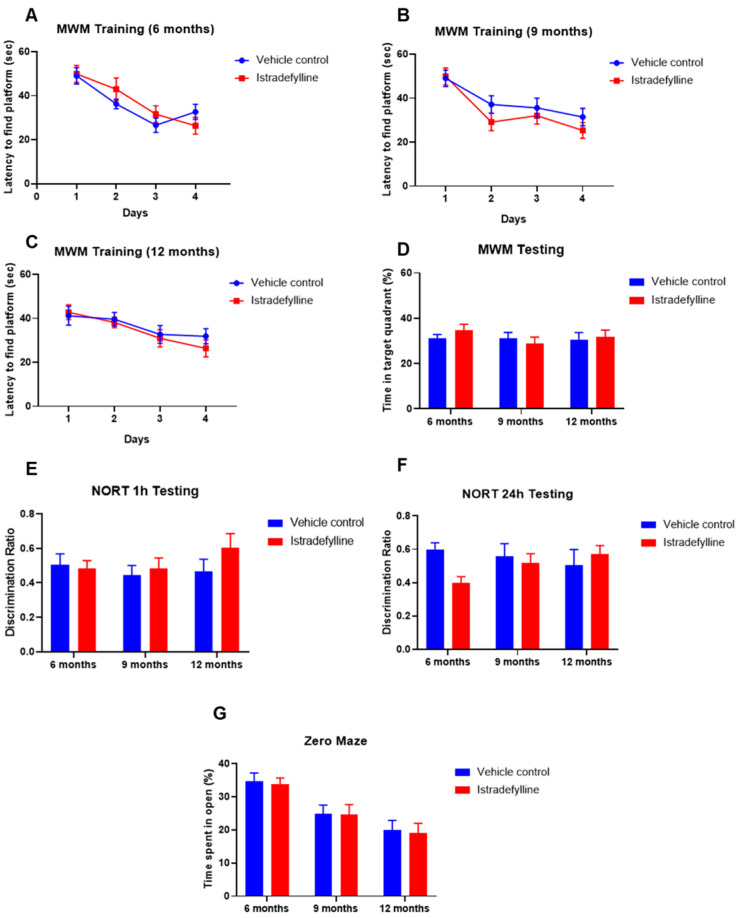
Cognitive studies in ageing mice. (**A**–**C**) Morris water maze (MWM) spatial memory training in istradefylline- and vehicle-treated mice at (**A**) 6 months, (**B**) 9 months, and (**C**) 12 months of age. (**D**) Final MWM testing in istradefylline-treated and control animals at 6, 9, and 12 months of age. Recognition memory was assessed by the novel object recognition test (NORT) after (**E**) 1 h (short-term memory) and (**F**) 24 h (long-term memory). (**G**) The level of anxiety in istradefylline-treated and control mice assessed by the zero maze test. Data presented as mean ± SEM. Vehicle control, *n* = 13; istradefylline, *n* = 12.

Behavioural studies were used to assess the effect of istradefylline on cognitive function and rule out neurotoxic side effects. The Morris water maze (MWM) was used to assess spatial learning and memory in ageing mice at the three established time points (6, 9, and 12 months of age). As expected, the latency to finding the platform was shorter with each day of training, but it was similar in control and istradefylline-treated mice (Figure 4A–C). When the platform was removed after four days of training (MWM testing), the amount of time spent in the target quadrant (previous location of the platform) was not affected by istradefylline treatment and was comparable at 6, 9, and 12 months of age (Figure 4D).

The novel object recognition test (NORT) was used to assess recognition memory. The time spent exploring the novel object and the familiar object was compared after 1 h (short-term memory) and 24 h (long-term memory). The discrimination ratio, calculated as the amount of interaction time with novel object divided by the total interaction with both objects, was assessed at 6, 9, and 12 months of age. The discrimination ratio did not change over time and the istradefylline-treated mice showed similar NORT outcomes in short- and long-term recognition memory as their vehicle-treated counterparts (Figure 4E,F).

The zero maze test was conducted at the same time points as the MWM and NORT to assess the anxiety in mice. The test shows that the mice tend to spend less time in the open section of the maze as they age, but their increased anxiety was not affected by istradefylline treatment (Figure 4G).

## 3. Discussion

Our study demonstrates the cochlear rescue effect of istradefylline treatment in ageing C57BL/6J mice, an established model of accelerated ARHL. The mice received weekly istradefylline injections for six months in middle age, with periodic measurements of auditory and cognitive functions at 6, 9, and 12 months. The chronic istradefylline treatment significantly reduced age-related threshold shifts (hearing loss) by about 20 dB at high frequencies (16 and 32 kHz), without affecting ABR suprathreshold responses. The improvement of ABR thresholds was supported by cochlear histology, as the istradefylline treatment yielded better preservation of the sensory hair cells. Cognitive function (spatial and recognition memory) remained largely unchanged in mice from 6 to 12 months of age. The level of anxiety increased in ageing mice; however, it was not affected by istradefylline treatment. The present study, thus, highlights the role of A_2A_R in the development of age-related cochlear degeneration in a mouse model of accelerated ARHL and suggests that istradefylline can partly preserve cochlear function and hearing in C57BL/6J mice without affecting cognitive function.

### 3.1. ABR Threshold Shifts and Hair Cell Loss in Istradefylline-Treated Mice

Chronic istradefylline treatment mitigated age-related ABR threshold shifts in mice at frequencies above 8 kHz at a level considered to be clinically significant (>10 dB) [28]. Auditory threshold shifts are largely determined by the integrity of sensory hair cells. At 12 months of age, we observed a significant loss in both types of sensory hair cells in the middle and basal segments of the cochlea in control vehicle-treated mice. OHC were more affected by ageing than IHC. Basal turn OHCs are particularly vulnerable to age-related degeneration, likely due to their lower antioxidant buffering capacity leading to increased susceptibility to oxidative stress [29]. Hair cell death is primarily induced by free radical-mediated oxidative stress and calcium overload, which in turn, leads to apoptosis in cochlear tissues [30]. In the present study, reduced auditory threshold shifts and improved hair cell survival in istradefylline-treated mice reflect the otoprotective effects of this drug.

### 3.2. ABR Suprathreshold Responses

Whilst auditory thresholds are considered a good metric of hair cell function, they are relatively poor indicator of neuronal damage in the cochlea [31]. In this study, the changes in wave I amplitude and latency were used as indicators of afferent neural fibre (ANF) integrity. Wave I suprathreshold responses were measured at frequencies corresponding to the low, middle, and high frequency regions of the cochlea (4, 16, and 32 kHz, respectively). Ageing significantly reduced wave I amplitudes and increased peak latencies in all test frequencies. This was likely due to reduction in synchronous firing, lower discharge rates, and decreased recruitment of ANFs [32]. The reduction in wave I amplitude corresponds to the age-related degeneration of the afferent IHC-auditory nerve synapses and spiral ganglion neurons [33,34]. As the treatment with istradefylline did not improve the ABR wave I suprathreshold amplitudes and latencies at any of the test frequencies, these findings support the idea that the beneficial effect of istradefylline on age-related auditory deficits in C57BL/6J mice is likely dominated by its effect on hair cell survival rather than cochlear neuropathy.

### 3.3. Cognitive Studies

Aging is associated with progressive changes in the brain and associated sensory, motor, and cognitive functions. Numerous studies in young and aged animals have reported differences in behavioural patterns suggesting age-related cognitive decline [14,35]. Here, we used well-established tests of spatial learning and memory (Morris water maze, MWM) [36], recognition memory (novel object recognition test, NORT) [37], and anxiety (zero maze test) [38] to investigate age-related changes in cognitive function and the effect of istradefylline. Our behavioural studies did not identify significant changes in cognitive function of C57BL/6J mice aged between 6 and 12 months; however, the anxiety increased in ageing mice. In the present study, istradefylline treatment did not improve spatial or recognition memory in C57BL/6J mice, but it also did not cause a decline in cognitive function, which is important when considering possible neurotoxic side effects of treatment.

### 3.4. Putative Mechanisms of Otoprotection by Istradefylline

Inhibition of the A_2A_R is a well-established mechanism of brain protection against adverse events such as ischemic brain damage, traumatic brain injury, excitotoxicity, and neurodegenerative conditions such as Parkinson’s and Alzheimer’s disease [20,39]. Antagonists of A_2A_R, including caffeine, can reverse memory impairments in aging rodents and in animal models of Alzheimer’s disease [40,41]. It was shown that the A_2A_R antagonists mitigate early onset cognitive dysfunction in mice after traumatic brain injury by reducing phosphorylation of tau proteins in the dentate gyrus [42]. Together, these studies established the strong therapeutic potential of A_2A_R inhibition in neurological diseases. Of particular interest for the present study is the clinically approved A_2A_R antagonist istradefylline, which has been used in patients with Parkinson’s disease (PD) along with L-DOPA to reduce cognitive and motor symptoms of PD [25,43]. The elimination half-life of istradefylline is 64–69 h after oral administration, which is the longest half-life of the available A_2A_ receptor antagonists [43]. In animal models of PD, istradefylline improved motor function by reducing A_2A_R’s inhibition of dopamine D2 receptor activity in GABAergic neurons of the striato-pallidal pathway [44].

The role of A_2A_R signalling in inner ear function in health and disease, however, is not well understood. A_2A_R are immunoexpressed in sensory hair cells, supporting Deiters’ cells and spiral ganglion neurons, but also in the spiral ligament fibrocytes and the modiolar blood vessels [45]. Immunolocalization of A_2A_R in diverse cochlear tissues suggests their role in various physiological processes in the cochlea, from sound transduction and auditory neurotransmission to cochlear blood flow. Previous studies have shown that activation of the A_2A_R with CGS-21680 in chinchilla cochlea can aggravate cisplatin-induced hearing loss [46], hinting at the possibility that inhibition of the A_2A_R may have an opposite effect. The cytoprotective effect of istradefylline in the present study is consistent with our previous work demonstrating excellent preservation of the sensory hair cells in A_2A_R-deficient mice after exposure to traumatic noise, which causes permanent SNHL in wildtype mice [23]. As the A_2A_R stimulation activates mitogen-activated protein (MAP) kinases [47], receptor inhibition may prevent apoptotic events induced by A_2A_R-mediated activation of MAP kinases (p38 and JNK1/2) in sensory hair cells.

An alternative explanation for the otoprotective effect of istradefylline in the ageing mouse cochlea is the altered balance between A_1_R and A_2A_R signalling pathways. It is well established that A_1_ and A_2A_ adenosine receptors operate in an opposing manner in the brain to regulate neuronal excitability and response to stress and injury [39], suggesting that the cytoprotective potential of adenosine receptor signalling can be based on stimulation of A_1_R and/or inhibition of A_2A_R. The present study demonstrates that istradefylline can partially rescue the cochlea from age-related degeneration by inhibiting A_2A_R signalling. Selective inhibition of the A_2A_R leads to preferential binding of endogenous adenosine to the A_1_R, which may reduce oxidative stress and lipid peroxidation in cochlear tissues by boosting endogenous antioxidant defences [48]. The A_1_R-dominant environment attained by selective inhibition of the A_2A_R may, in theory, counteract the underlying mechanisms of age-related cochlear injury, including oxidative stress, calcium overload, and glutamate excitotoxicity [49]. Indeed, the altered receptor balance improved the survival of sensory hair cells in the cochlea and mitigated ARHL in ageing C57BL/6J mice. However, istradefylline treatment did not improve the survival of afferent synapses and ANF in the mouse cochlea, as the suprathreshold responses were similar in control and istradefylline-treated mice. This was unexpected, as our previous study in cochlear tissue cultures demonstrated excellent preservation of IHC-auditory nerve synapses and ANF after excitotoxic injury in tissues treated with istradefylline [24]. However, that study [24] was performed in organotypic tissue cultures of the neonatal rat cochlea; hence, it is difficult to draw parallels with the present study carried out in adult mice.

## 4. Materials and Methods

### 4.1. Animals

Six-month-old male C57BL/6J mice were used in this study. The mice were housed under controlled conditions (constant humidity and temperature, 12 h light/dark cycle) with free access to food and water at the University of Auckland animal facility for the duration of the study (6 months). At the beginning of the study, the mice were weighed and checked for general health indicators (appearance and behaviour). Animal welfare was continuously assessed throughout the study to ensure animal health was maintained at the highest standard. A total of 25 mice were included in this study: 12 mice were treated with istradefylline, and 13 control mice were injected with an equivalent volume of the drug vehicle solution. Weekly injection of istradefylline or drug vehicle commenced at 6 months and were carried out until 12 months of age. All experimental procedures described in this study were approved by the University of Auckland Animal Ethics Committee.

### 4.2. Auditory Brainstem Responses 

Auditory thresholds were evaluated in all mice at 6 months (baseline), 9 months, and 12 months of age (final) using auditory brainstem responses (ABR). The researcher performing ABR measurements and data analysis was blinded to treatment to eliminate potential bias in threshold assessment.

ABR are auditory evoked potentials that represent the summation of electrical activity in the auditory nerve and central auditory pathways in response to auditory clicks and tone pip stimuli. ABRs were recorded in a sound attenuating chamber (Shelburg Acoustics, Sydney, Australia) equipped with internal ventilation, a light source, and a thermostatically controlled electric blanket. The mice were anaesthetised with intraperitoneal injections of ketamine (100 mg/kg) and xylazine (10 mg/kg). The external auditory canal and tympanic membrane were checked for signs of infection, physical trauma, or scarring prior to ABR recordings. Three subdermal platinum electrodes connected to a Medusa RA4LI headstage amplifier (×20 gain) were used to record ABR responses. The reference electrode was placed at the mastoid region of the left ear, the active electrode at the scalp vertex, and the ground electrode at the mastoid region of the right ear. The Tucker-Davis Technologies (TDT) auditory physiology System III workstation and BioSig digital signal processing software (Alachua, FL, USA) were used to generate the acoustic stimuli and to record the ABR. A multi-field magnetic speaker (MF1, TDT) with a plastic sound delivery tube was used to deliver auditory stimuli into the external auditory canal of the left ear. A series of tone pip stimuli (5 ms duration, 1.5 ms rise/fall times, 4–32 kHz) were presented to elicit ABR responses. Tone pip responses were acquired at an alternating polarity sampling rate of 512 and averaged for each sound intensity. A bandpass filter of 300–3000 Hz, 50 Hz notch was applied to all responses. At the end of the recording, mice were given a subcutaneous injection of antisedan (1 mg/kg) to reverse the effects of ketamine–xylazine anaesthesia.

The ABR wave I was used to determine auditory thresholds defined as the lowest sound intensity level capable of eliciting a reproducible waveform. The cut off amplitude was set at =125 nV, as consistently reproducible waveforms were obtained at and above this amplitude. Repeat waveforms were analysed at each frequency to determine the consistency of the responses and to identify the recurring peaks. In cases where the threshold ceiling was exceeded (thresholds above 90 dB SPL), thresholds were arbitrarily assigned a value of 95 dB SPL.

The amplitudes and latencies of wave I for frequencies 4, 16, and 32 kHz were assessed at suprathreshold intensities to investigate the effect of istradefylline on the development of age-related auditory nerve degeneration. The amplitude of wave I (peak to trough) was measured at 80, 85, and 90 dB SPL intensities, and latency was measured as the time taken to reach the peak (including 0.3 ms signal transduction time from the speaker to the ear).

### 4.3. Istradefylline Treatment

Drug solution was made by dissolving istradefylline (Sigma Aldrich, St. Louis, MO, USA) in 2% DMSO and 0.9% saline, then, aliquoted and stored at −20 °C. Istradefylline was injected to wild-type C57BL/6J mice intraperitoneally once a week at a dose of 1 mg/kg, commencing at the age of 6 months until the age of 12 months. An equivalent volume of the drug vehicle solution was administered by intraperitoneal injection to the control group of mice once a week.

### 4.4. Cochlear Tissue Preparation

After the final ABR assessment, animals were euthanized by an anaesthetic overdose (pentobarbitone, 100–150 mg/kg intraperitoneally). The animals were perfused through the heart with the flush solution (0.9% NaCl containing 10% NaNO_2_) followed by whole body perfusion with 4% paraformaldehyde (PFA) in 0.1 M phosphate buffer (PB, pH 7.4). The cochleae were dissected from the temporal bone and fixed in 4% PFA overnight at 4 °C and, then, decalcified using 5% EDTA in 0.1 M PB for 9 days at 4 °C.

### 4.5. Hair Cell Counting

Cochleae were decapsulated and the organ of Corti removed and dissected into apical, middle, and basal turns, with particular care taken to remove the lateral wall, Reissner’s membrane, and tectorial membrane. Microdissected cochlear turns were transferred to a 48-well plate containing 0.1 M PBS (pH 7.4), permeabilised with 1% Triton X-100 in 0.1 M PBS (pH 7.4), and blocked with 10% normal goat serum (NGS) for 2 h at room temperature (RT). Wholemount tissues were, then, incubated overnight at 4 °C with the rabbit polyclonal anti-Myosin VIIa antibody (Proteus Biosciences, 1:500) in solution containing 0.1% Triton X-100 in 0.1 M PBS with 5% NGS. The following day, wholemounts were washed four times for 60 min with 0.1 M PBS and, then, incubated for 2 h in the dark at RT with the goat anti-rabbit IgG (Alexa 568, 1:500; Invitrogen) in antibody solution containing 0.1% Triton X-100 in 0.1 M PBS with 5% NGS. Wholemounts were, then, washed for 60 min with 0.1 M PBS and mounted on glass slides using Citifluor AFI mounting medium, cover-slipped, sealed with nail polish, and stored in the dark at 4 °C.

For hair cell counting, each cochlea was divided into three segments covering the entire length of the cochlea and representing different frequency regions [50]. The apical segment occupied approximately 0–30% distance from the apex, middle segment 30–65% distance from the apex, and basal segment 65–100% distance from the apex. Inner and outer hair cell counts were determined for the apical, middle, and basal turns of the istradefylline-treated and vehicle-treated cochleae. The organ of Corti was imaged with a Zeiss Axioplan 2 epifluorescence microscope (Carl Zeiss, Jena, Germany) with 20× objective (0.6 NA) and captured with a Photometrics Prime sCMOS monochrome camera. Hair cells were counted in ImageJ using the CellCounter plugin to mark intact and missing hair cells, with the number of missing cells expressed as a percentage of the total number of hair cells counted. For regions with complete OHC loss, but intact IHC, one IHC was approximated to three missing OHC (one in each row). For regions of absolute hair cell loss, an adjacent length of IHC was measured (3–4 cells) to calculate pixel width per IHC. The distance of the lesion was measured, and the number of missing IHC and corresponding missing OHC was estimated.

### 4.6. Morris Water Maze

Morris water maze (MWM) is a widely used behavioural procedure for the assessment of spatial learning and memory in small rodents. The MWM test apparatus consists of a 130 cm-diameter-wide black circular pool filled with opaque tap water maintained at 22 °C with added non-toxic white dye. A circular hidden escape platform (10 cm in diameter) just beneath the surface was placed within the pool surrounded by four elevated visual navigation cues (coloured geometric images) above each pool quadrant to provide spatial orientation. The training was performed under the red LED lighting as rats are unable to see that colour. The mice were trained to find the platform placed in one quadrant on their first trial followed by additional three trials at 24 h intervals. On each acquisition day, four test runs were conducted for each mouse with a resting time in between. The mice were dried using towel and kept in recovery cage with heat lamp, and a minimum of half an hour resting period was provided after each trial. The starting position for every trial was assigned randomly. The location of the platform was kept consistent, except on day 5, and the latency to finding the platform was measured and plotted over the four days. If the platform was not found, the time was plotted as 60 s. On day 5 (the test day), the platform was removed, and two trials (60 s each) were conducted under indirect LED lighting to record the amount of time spent in the target quadrant where the platform was previously located. The tracking system EthoVision (Noldus, Wageningen, The Netherlands) was used to determine the latency to find the platform (MWM training) and the time spent in the target quadrant (MWM testing). Both the training and testing sessions were performed in daytime (9 a.m.–5 p.m.).

### 4.7. Novel Object Recognition Test

The novel object recognition test (NORT) evaluates a rodent’s ability to recognise a novel object in the environment, without any positive or negative enforcement. When rodents are exposed to a familiar and a novel object at the same time, they approach the novel object more frequently and spend more time exploring it than the familiar object. In this study, NORT was performed to examine the short-term (1 h) and long-term (24 h) recognition memory in mice. The amount of time taken to explore the new object provides an index of recognition memory. The mice were habituated to an in-house built plexiglass arena measuring 25 cm × 29 cm × 25 cm twice a day (15 min each trial) with a minimal resting time of 2 h, for 3 days. On day 4 (NORT testing), the mice were placed in the centre of the box containing two identical objects and allowed to explore for 5 min. After 1 or 24 h, one of the objects was replaced by a novel object, and the time spent exploring the novel object and the familiar object was recorded using the EthoVision tracking system. Exploration was defined as the time spent with the head oriented towards and within 2 cm of the object. Standing, sitting, or leaning on the object was not considered as exploration. The discrimination ratio was calculated as the amount of interaction time with novel object divided by the total interaction time with both objects. Low discrimination ratio indicates impaired recognition memory.

### 4.8. Zero Maze Test

The zero maze test was conducted at the same age as other behavioural tests (6, 9, and 12 months) to assess the level of anxiety in mice. The zero maze apparatus consists of an in-house made opaque plexiglass circular platform (diameter 40 cm) elevated 40 cm above the ground, divided equally into two closed and two open sections. Mice have a natural tendency to avoid heights, and anxious animals spend more time in the closed sections, which is seen as a sign of increased anxiety in open and elevated spaces. The mice were placed in the closed section followed by 5 min of movement recording using the EthoVision tracking system. The percentage of total time spent in open section was compared between istradefylline- and vehicle-treated animals.

### 4.9. Data Analysis

The researchers were blinded for all ABR assessments, tissue collections, histological, and behavioural analyses. Animals were assigned a subject ID by an independent researcher and allocated into the treatment or vehicle control group using a randomly generated number list (https://www.randomizer.org, accessed on 11 July 2019). Aliquoted injection solutions were labelled only by subject ID. All data were tested for normality using the Shapiro–Wilk Test. Auditory thresholds, cognitive function, and hair cell counts were analysed using a two-way ANOVA with a post hoc Holm–Sidak test. Suprathreshold data were analysed using multi-level factorial ANOVA with planned orthogonal contrasts to determine differences between groups. Data are presented as mean ± SEM.

## 5. Conclusions

This study demonstrates a robust rescue effect of istradefylline against age-related cochlear degeneration in a mouse model of ARHL. Further studies are required to establish the mechanism of cochlear protection by istradefylline and determine whether istradefylline has a preventative or therapeutic role in age-related cochlear degeneration. Other constraints include a dose-response study, drug distribution in the cochlea after systemic administration and investigation of the optimal therapeutic time window for istradefylline treatment. Translational studies should aim to expand on the clinical potential of this treatment in the therapeutic management of ARHL and related cochlear sensorineural pathologies.

## Data Availability

Not applicable.
